# Molecular Characterization and Phylogenetic Analysis of Serotype 8b Fowl Adenoviruses from Commercial Broiler and Layer Flocks with Hepatitis

**DOI:** 10.3390/v18040415

**Published:** 2026-03-27

**Authors:** Ozge Ardicli, Tugce Serim Kanar, Juon Abbass, Mehmet Ekik, İpek Colak Budak, Melek Z. Demirci, Serpil Kahya Demirbilek, Huseyn Babayev, K. Tayfun Carli

**Affiliations:** 1 Milk and Dairy Products Technology Program, Division of Food Processing, Karacabey Vocational School, Bursa Uludag University, Bursa 16700, Türkiye; ozgeyilmaz@uludag.edu.tr; 2 Department of Microbiology, Faculty of Veterinary Medicine, Bursa Uludag University, Bursa 16059, Türkiye; tugcesrm@gmail.com (T.S.K.); abbasjohn205@gmail.com (J.A.); ipekcolak00@gmail.com (İ.C.B.); melekzubeydedemirci@gmail.com (M.Z.D.); serpilkahya@uludag.edu.tr (S.K.D.); 3 Makrovet Veterinary Analysis Laboratory, Karatay, Konya 42050, Türkiye; m.ekik@makroveterinerlik.com.tr; 4 Swiss Institute of Allergy and Asthma Research (SIAF), University of Zurich, 7265 Davos, Switzerland; babayev@silicosome.com

**Keywords:** Fowl adenovirus (FAdV), serotype 8b, inclusion body hepatitis (IBH), hexon gene

## Abstract

This study investigated the molecular characteristics and genetic diversity of Fowl adenovirus (FAdV) strains circulating in commercial broiler and layer flocks in the Southern Marmara and Aegean regions of Türkiye between January and December 2025. Liver samples (*n* = 120) collected from twelve flocks with increased mortality and clinical signs compatible with adenoviral infection were analyzed. Detection was performed using circular amplification technology and PCR targeting the hexon L1 region, and positive samples were sequenced for molecular characterization. BLAST analysis showed that all isolates belonged to *Aviadenovirus hepatitidis* and were identified as serotype 8b. Pairwise comparisons showed high nucleotide identity among isolates (97.4–100%) and 98.1–100% similarity with the Turkish reference strain MK937075. Only three isolates displayed nucleotide substitutions, while most sequences were identical within the analyzed region. Amino acid similarity ranged from 95.2% to 100%. Phylogenetic analysis revealed that all isolates clustered within a single monophyletic group together with previously reported Turkish FAdV-8b strains. Necropsy findings included hepatomegaly, multifocal hepatic pallor, petechial hemorrhages, gizzard erosion, and serous pericardial involvement. The detection of genetically closely related isolates across multiple provinces suggests regional circulation of a common viral lineage. These findings demonstrate that FAdV-8b is currently the predominant serotype associated with inclusion body hepatitis outbreaks in this major poultry production area and highlight the importance of molecular surveillance and targeted control strategies, including breeder monitoring and region-specific vaccine development.

## 1. Introduction

Fowl adenoviruses (FAdVs) infect a wide range of avian species, particularly chickens, and are important pathogens in the poultry industry worldwide due to their association with significant economic losses [1]. These viruses are associated with subclinical cases to systemic disease, most notably inclusion body hepatitis (IBH), hepatitis-hydropericardium syndrome (HHS), and adenoviral gizzard erosion (AGE), all of which can result in substantial productivity losses due to mortality, reduced weight gain, and impaired flock performance. Transmission occurs through both vertical and horizontal routes. High fecal viral loads facilitate environmental dissemination and persistence within poultry facilities, thereby increasing infection risk in commercial operations [1,2].

FAdVs are non-enveloped, icosahedral, double-stranded DNA viruses belonging to the genus *Aviadenovirus* within the family *Adenoviridae*. Their genome is approximately 43–45 kb in length and coding for 10 primary structural proteins and 11 nonstructural proteins [3]. The capsid structure of FAdVs is composed primarily of three major proteins, hexon, fiber, and penton, which serve as critical markers for both pathogenesis and molecular epidemiology. Among these, the hexon gene is especially important because it encodes the largest and most immunodominant capsid protein of adenoviruses, forming the icosahedral capsid’s facets and containing antigenic determinants responsible for serotype specificity. Hence, the hexon protein is used for genotyping and classification [1,4,5,6].

FAdVs are classified into five species that infect chickens: *Aviadenovirus ventriculi*, *Aviadenovirus quintum*, *Aviadenovirus hydropericardii*, *Aviadenovirus gallinae*, and *Aviadenovirus hepatitidis*, which correspond to the previously defined species groups (A-E) [7,8]. These viruses are further divided into 12 serotypes (FAdV-1 to FAdV-11, including 8a and 8b) by cross-neutralization assays. In addition, analyses of hexon gene sequences have identified at least 12 genotypes within these species, highlighting the considerable genetic diversity of circulating strains [1]. Distinct species and serotypes are associated with specific disease manifestations. For example, FAdV-2 and FAdV-11 (*A. gallinae*) and FAdV-8a and FAdV-8b (*A. hepatitidis*) are most commonly linked to IBH, whereas HHS is primarily attributed to FAdV-4 (*A. hydropericardii*). Moreover, FAdV-1, belonging to *A. ventriculi*, is frequently isolated from cases of AGE [9]. This diversity in species distribution and disease association highlights the importance of accurate molecular classification for epidemiological surveillance, diagnosis, and the development of effective prevention strategies.

The high prevalence and genetic variability among circulating strains have remarkably increased the global impact of FAdV-associated diseases in poultry. Despite the publication of molecular studies on adenoviruses in recent years [3,10], it is evident that the samples analyzed in many of these investigations were collected several years earlier, typically 4–5 years prior to publication. The capacity of FAdVs to generate emerging strains underscores the necessity of continuous surveillance and ongoing molecular characterization to accurately monitor viral evolution and epidemiological dynamics. The present study aimed to detect and genotypically characterize FAdV strains circulating in commercial poultry flocks in the Southern Marmara and Aegean regions of Türkiye from January to December 2025 and to evaluate their molecular diversity to improve epidemiological understanding and support the development of targeted control strategies.

## 2. Materials and Methods

### 2.1. Ethical Statement

This study was conducted in accordance with national and institutional ethical guidelines. All samples were collected during routine veterinary post-mortem examinations of commercial poultry flocks experiencing natural disease outbreaks.

### 2.2. Study Area and Population

The study was conducted from January to December 2025 in the Southern Marmara and Aegean regions of Türkiye, a major hub for the country’s poultry industry. Sampling targeted commercial broiler and layer flocks in the provinces of Canakkale, Balikesir, Bursa, and Afyon. The study population consisted of 10 commercial Ross broiler flocks and two Lohmann Lite layer flocks. The affected birds were 15–38 days old in broilers and 18 weeks old in layers. These flocks were selected based on reports of sudden increases in mortality and clinical signs indicative of Adenovirus infection. The recorded mortality rates in these flocks ranged from 3.4% to 10.5%.

### 2.3. Sample Collection and Necropsy

A total of 120 tissue samples were collected from the 12 affected flocks. Systematic necropsies were performed on birds that had recently died or were culled in an agonized state. During post-mortem examination, gross pathological lesions were recorded, with particular attention paid to the liver, heart, gizzard, and bursa of Fabricius. Liver samples were aseptically collected, pooled by flock (10 organs per pool), placed in sterile containers, and transported to the laboratory under a cold chain (+4 °C) for subsequent molecular detection and characterization.

### 2.4. Nucleic Acid Extraction

Approximately 25 mg of liver tissue was homogenized using a tissue homogenizer (MagNA Lyser Rotor Roche Diagnostics, Almere, The Netherlands). The homogenate was transferred into tubes containing Molecular Transport and Lysis Reagent (MTRL; Nucleogene Biotechnology Co., Istanbul, Türkiye) and incubated for 1 h at room temperature. After incubation, the supernatant was transferred to a spin column placed in a collection tube and then centrifuged at 8000× *g* for 1 min. Subsequently, 500 µL of 80% ethanol was added to the column, and the mixture was centrifuged again at 8000× *g* for 1 min. The column was then centrifuged at 16,000× *g* for 1 min to remove residual ethanol. Nucleic acids were eluted by adding 50 µL of nuclease-free water to the center of the spin column, followed by centrifugation at 8000× *g* for 1 min. The concentration and purity of the extracted DNA were assessed using a spectrophotometer (NanoDrop™, Thermo Fisher Scientific, Wilmington, DE, USA). The eluted nucleic acids were stored at −80 °C until further analysis.

### 2.5. Molecular Detection

Initial screening for FAdV was performed using a molecular detection assay based on circular amplification technology (MDA-CAT; Nucleogene Biotechnology Co., Istanbul, Türkiye) to rapidly identify positive flocks. Positive samples were further subjected to PCR for genotypic characterization. The PCR targeted the L1 variable region of the hexon gene (590 bp). The primers used were based on the protocol described by Mase et al. (2009) [11]. PCR amplification was performed in a total volume of 25 μL containing 2.5 μL of template DNA, 12.5 μL of 2× PCR Master Mix (Qiagen, Hilden, Germany), 0.5 μL of each primer (10 μM), and 9 μL of nuclease-free water. The thermal cycling conditions were initial denaturation at 94 °C for 5 min; followed by 35 cycles of denaturation at 94 °C for 30 s, annealing at 55 °C for 30 s, and extension at 72 °C for 30 s; with a final extension at 72 °C for 5 min. Amplified products were purified and sequenced using an automated DNA sequencer (ABI PRISM 310 Genetic Analyzer, Applied Biosystems, Waltham, MA, USA).

### 2.6. Phylogenetic Analysis

The obtained sequences were analyzed alongside reference FAdV strains retrieved from the National Center for Biotechnology Information (NCBI) GenBank database. Multiple sequence alignments were performed, and phylogenetic analyses were conducted using MEGA version 11. Phylogenetic inference was conducted using the Maximum Likelihood (ML) method implemented in IQ-TREE version 2.4.0. The K3Pu + F + G4 nucleotide substitution model was applied, as identified by ModelFinder as the best-fit model. To maximize topological accuracy, an exhaustive Nearest Neighbor Interchange (NNI) search was enforced with a stopping criterion of 500 iterations. Node support was evaluated using 1000 replicates for both the SH-like approximate likelihood ratio test (SH-aLRT) and the Ultrafast Bootstrap approximation. The final tree was rooted with FAdV-11 (MK937072.1) as the outgroup. The phylogenetic tree was generated by comparing the sequences of the hypervariable loop regions of the hexon protein compared with selected reference strains (MK937072.1 TR/BVKE/R/AC-2 Türkiye; NC 038332.1 CR119 USA; OR901942.1 SDQD/2023 China; NC 075459.1 TR59 Türkiye; KC750801.1 4350/2011 Debrecen Hungary; PP153927.1 UPM T27 Malaysia; NC 075460.1 YR36 China; MF577036.1 QD2016 China; MG712775.1 SD1356 China; OK188966.1 HeB20 China; PP537790.1 HD2402 China; NC 075458.1 764 United Kingdom; MK937076.1 TR/BVKE/R/Y Türkiye; KC750780.1 38259/2009 Debrecen Hungary; KU517714.1 UPM04217 Malaysia; OL456208.1 SD2009 China; MK937075.1 TR/BVKE/R/D-1 Türkiye) [12,13,14].

### 2.7. Structural Modeling

The 3D model of the FAdV-8b hexon protein was predicted from its reference amino acid sequence (GenBank: YP_004191821.1) using AlphaFold 3. Generated atomic coordinates were imported into UCSF ChimeraX for visualization and structural analysis. Lineage-specific amino acid substitutions were mapped onto the hexon ribbon backbone and rendered as color-coded atomic spheres to evaluate topological distribution and spatial clustering across the L1 region.

## 3. Results

### 3.1. Clinical History and Necropsy Findings

**Figure 1:**
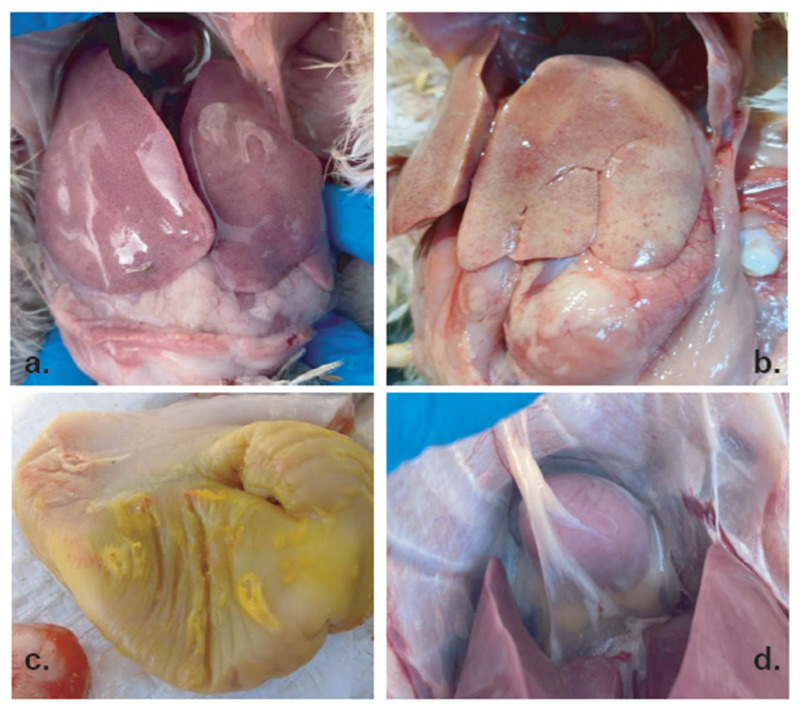
Gross pathological lesions observed in broiler chickens (15–38 days old) infected with FAdV. (**a**,**b**) Enlarged liver showing pale discoloration and multifocal lesions; (**c**) gizzard mucosa with yellow necrotic material and detachment of the koilin layer; (**d**) serous fluid accumulation on the surface of the heart.

In broilers and layers, disease onset occurred at 15–38 days of age and 18 weeks of age, respectively. Mortality rates varied among flocks, ranging from 3.4% to 10.5%. Clinical observations indicated depression, lethargy, and a sudden increase in flock mortality. Gross pathological examination revealed consistent lesions primarily affecting the liver, gizzard, and heart (Figure 1). The liver was markedly enlarged and showed a pale reddish-brown to tan discoloration, with multifocal, irregular pale areas (Figure 1a,b). Multiple petechial hemorrhages were also evident on the hepatic surface. Examination of the gizzard demonstrated yellow necrotic material on the mucosa, accompanied by detachment of the koilin (keratinoid) layer (Figure 1c). Additionally, serous exudate was observed on the surface of the heart (Figure 1d). All gross lesions were consistent across affected flocks and were characterized by hepatomegaly with multifocal hepatic pallor, gizzard erosion, and serous pericardial involvement.

### 3.2. Molecular Detection and Genotypic Characterization

Nucleotide sequencing was successfully performed for all pooled tissue samples. BLAST (https://blast.ncbi.nlm.nih.gov/Blast.cgi (accessed on 22 March 2026)) analysis of the obtained sequences against the NCBI GenBank database revealed that all field isolates belonged to *A. hepatitidis* and were identified as serotype 8b. Pairwise sequence comparison showed a high level of nucleotide identity among the isolates (97.4–100%; Table 1), suggesting the circulation of a closely related viral population and indicating either a common infection source or the predominance of a single strain in the Southern Marmara and Aegean regions during the study period.

Comparative nucleotide analysis with the reference strain TR/BVKE/R/D-1 (MK937075) revealed very high sequence similarity among all isolates (98.1–100%). Only three isolates exhibited nucleotide substitutions relative to the TR/BVKE/R/D-1 (MK937075) sequence. Six substitutions were detected in FAdV_Broiler_Turkiye_4 (g.566 G>A; g.568 G>A; g.598 G>C; g.620 G>T; g.636 G>A; g.638 G>A), whereas FAdV_Broiler_Turkiye_2 and FAdV_Broiler_Turkiye_10 showed one (g.636 G>A) and two substitutions (g.356 A>C and g.358 C>A), respectively (Table 1). All remaining isolates were identical to the reference sequence within the analyzed region.

Analysis of deduced amino acid sequences of the hexon L1 loop region demonstrated strong protein conservation. Most isolates showed complete amino acid identity with MK937075. Only two isolates displayed substitutions: FAdV_Broiler_Turkiye_4 contained five amino acid changes (S189N, E190K, A200P, R207L, S213N), whereas FAdV_Broiler_Turkiye_10 exhibited two substitutions (K119T and P120T) (Table 2). Amino acid similarity ranged from 95.2% to 100%, indicating limited variability within the analyzed hexon region among circulating viruses.

### 3.3. Phylogenetic Evaluation

**Figure 2:**
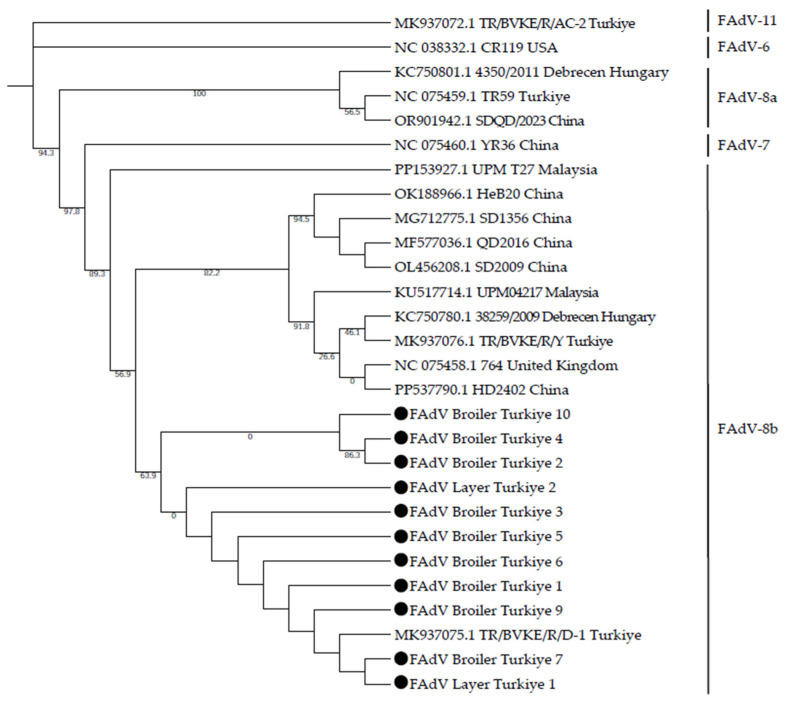
Phylogenetic tree constructed using the Maximum Likelihood method based on partial hexon gene sequences (316 bp). Turkish isolates obtained in this study are marked with black circles. Reference sequences (*n* = 17) were retrieved from the GenBank database. The scale bar represents nucleotide substitutions per site.

Phylogenetic analysis based on hexon gene sequences showed that all 11 Turkish isolates clustered within the FAdV-8b lineage (Figure 2). In the phylogenetic tree, the study isolates formed a single, well-supported monophyletic cluster together with the Turkish reference strain MK937075.1 (TR/BVKE/R/D-1). Within this cluster, most isolates are grouped tightly with minimal branch divergence, indicating very close genetic relatedness. Two isolates (FAdV_Broiler_Turkiye_4 and FAdV_Broiler_Turkiye_2) formed a slightly separated sub-branch but remained within the same FAdV-8b cluster. The Turkish cluster showed close phylogenetic proximity to previously reported international FAdV-8b strains, including isolates from China, Malaysia, Hungary, and the United Kingdom, which were positioned within the same major clade. In contrast, representative sequences of other serotypes (FAdV-7, FAdV-6, FAdV-8a, and FAdV-11) formed clearly distinct branches, confirming the serotype classification of the study isolates and demonstrating their separation from non-8b lineages.

## 4. Discussion

Fowl adenovirus infections are a growing concern for the global poultry industry due to their diverse clinical manifestations, economic impact, and evolving molecular epidemiology [8,15,16]. While FAdV infections have been documented in Türkiye since 2019, understanding the evolving epidemiology of circulating serotypes is crucial for effective control [17]. The present study provides a comprehensive molecular and pathological characterization of FAdV outbreaks in commercial broiler and layer flocks from the Southern Marmara and Aegean regions, two major poultry production centers in Türkiye. The detection of FAdV in all examined flocks, combined with mortality rates ranging from 3.4% to 10.5%, confirms the active circulation of pathogenic FAdV strains in this region.

In this study, molecular characterization revealed that all isolates belonged to the *A. hepatitidis* and were serotype 8b. One of the earliest reports by Şahindokuyucu et al. (2020) [17] identified both FAdV-8b and FAdV-11 as causative agents of IBH in the Aegean region between January and March 2019. Similarly, Cizmecigil et al. (2020) [18] characterized FAdV-8b outbreaks in 8-day-old broilers in the same region, noting high mortality (10–14%) and severe hepatitis. However, a recent comprehensive longitudinal study covering 2020–2021 indicated a significant epidemiological shift towards the dominance of FAdV-8b, which accounted for nearly 95% of isolates (55 out of 58) compared to FAdV-11 [10]. Our findings of the sampling period support this trend and suggest that FAdV-8b has become the principal circulating serotype in western regions of the country. This epidemiological transition may reflect selective advantages such as enhanced transmissibility, environmental persistence, or adaptation to local poultry production systems.

An additional epidemiological observation of particular interest was that all isolates obtained from geographically distinct production systems belonged to the same serotype (FAdV-8b). Layer flocks originated from the Aegean region (Afyon), whereas broiler flocks were in the Southern Marmara region. Despite these spatial and production-type differences, the detection of a single serotype with high genetic similarity across all isolates suggests the possible circulation of a closely related viral population.

Phylogenetic analysis further supported the molecular findings by demonstrating that all isolates clustered within a single monophyletic group together with previously reported Turkish FAdV-8b strains. The hexon gene was selected for phylogenetic analysis because it encodes the largest capsid protein and contains serotype-specific antigenic determinants responsible for viral classification [5]. This gene is widely regarded as the principal marker for molecular typing of FAdVs, as sequence variability in its hypervariable loop regions enables reliable genotype differentiation and supports epidemiological surveillance. Comparative sequence analysis revealed variability within this region. Two isolates harbored missense mutations, and one isolate exhibited a silent mutation relative to the Turkish reference strain, whereas the remaining isolates were identical. Although only a few mutations were detected, variations in this region may be biologically significant because it contains antigenic sites that elicit protective antibodies; even limited substitutions could induce conformational changes affecting antigenicity. Zhang et al. (2021) [19] showed that arginine-to-isoleucine at position 188 in FAdV-4 hexon induces conformational shifts affecting antibody neutralization and pathogenicity.

**Figure 3:**
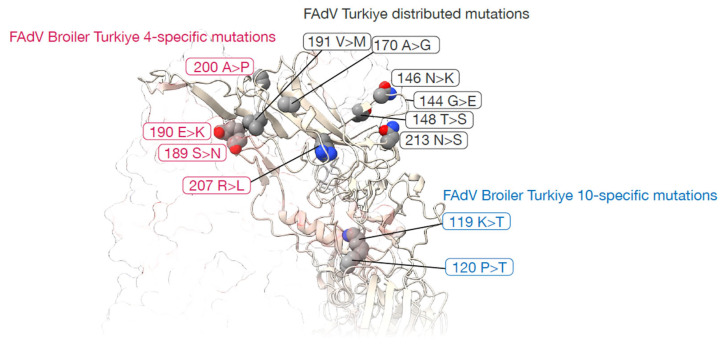
Structural mapping of FAdV-8b hexon mutations. Widespread Turkish FAdV-8b substitutions (gray; 144 G>E, 146 N>K, 148 T>S, 170 A>G, 191 V>M, 213 N>S) map across the exposed hexon loop-1 (L1), indicating selective pressure for immune evasion. Türkiye 4-specific mutations (pink; 189 S>N, 190 E>K, 200 A>P, 207 R>L) cluster tightly within the L1 hypervariable region, likely modifying type-specific neutralizing epitopes. Türkiye 10-specific substitutions (blue; 119 K>T, 120 P>T) localize at the L1 base near the Pedestal 1 (P1) junction, potentially modulating the structural flexibility of the hexon trimer.

The spatial clustering of broadly distributed and Türkiye 4-specific mutations within the exposed Hexon Loop-1 (L1) suggests a potential role in antigenic drift, possibly driven by host immune pressure. Given that the L1 region typically encompasses major neutralizing epitopes, it is plausible that these substitutions might contribute to immune evasion. Conversely, the distinct localization of Türkiye 10-specific mutations near the pedestal junction raises the possibility of alternative evolutionary dynamics, perhaps influencing hexon trimer stability or capsid assembly rather than directly altering antibody binding (Figure 3). Ultimately, these structural variations hint at the ongoing adaptation of circulating Turkish strains, highlighting a need for further in vitro and in vivo studies to determine their true biological significance.

Gross pathological liver findings observed in this study were consistent with typical lesions reported in FAdV-associated disease, including hepatomegaly, pale yellow livers, and petechial hemorrhages, which were consistent with descriptions from previous Turkish outbreaks [17,18] and global reports of FAdV-8 infections [20,21]. However, additional lesions, including pericardial effusion and gizzard erosion, were also detected. Although AGE is classically attributed to FAdV-1, an experimental study has demonstrated that serotype 8 strains can also induce gizzard lesions [22]. In addition, traditionally, hydropericardium syndrome is associated with FAdV-4 [2]. The presence of these lesions in birds infected exclusively with FAdV-8b suggests that this serotype may possess a broader pathogenic potential than previously assumed, possibly influenced by strain-specific virulence traits or host-related factors.

On a global scale, the dissemination and clinical impact of FAdV infections are shaped by interconnected epidemiological risk factors. Foremost among these is vertical transmission, which plays a decisive role in maintaining disease within production systems. Infected breeder hens transmit the virus through eggs to their progeny, thereby seeding infection in commercial flocks from the first day of life and rendering eradication nearly impossible without effective intervention at the breeder level [23,24,25]. Following vertical introduction, horizontal transmission occurs highly efficiently. Infected birds shed large quantities of virus, leading to fecal-oral spread and contamination of litter, water, and feed. Moreover, the non-enveloped structure of FAdVs enhances their environmental stability, allowing the virus to persist for weeks on fomites such as crates, transport vehicles, and equipment, thereby facilitating mechanical spread between farms [1,26].

It has been widely reported that FAdVs belonging to different genotypes are circulating globally and causing substantial economic losses in commercial poultry flocks. From an evolutionary perspective, dominant variants may undergo continuous genetic change driven by mutation, recombination, and selection pressures, thereby altering their epidemiological and pathogenic characteristics. This dynamic nature of viral evolution underscores the necessity for continuous surveillance and systematic monitoring programs. The generation of updated regional datasets and comprehensive research reports is therefore essential to accurately track emerging variants, understand shifting genotype dominance, and implement timely control strategies. Currently, Asia is regarded as the principal hotspot for FAdV activity and the emergence of new variants [27]. In Europe, FAdV infections are considered endemic, with IBH representing the predominant clinical manifestation. In this region, serotypes FAdV-8b and FAdV-11 are most frequently reported and have been extensively documented in countries such as Poland, Hungary, and Austria [5,28,29]. These geographic differences indicate that FAdV epidemiology is closely linked to regional production models, flock density, and biosecurity practices [27].

In recent years, the understanding of FAdV epidemiology has changed substantially. Viruses once regarded as secondary or minor pathogens are now recognized as major and persistent infectious agents worldwide in poultry. This global expansion is closely associated with the intensification of poultry production and the virus’s capacity for ongoing genetic diversification. Although the overall global distribution of FAdVs appears comparable across major poultry-producing regions, the predominant serotypes and associated clinical presentations vary between countries. These differences are likely driven by the interplay between viral genetic characteristics, host susceptibility, and management practices [1,2].

Considering the cosmopolitan distribution of FAdV infections, these viruses are currently recognized among the most economically significant viral pathogens affecting the poultry industry worldwide. Molecular epidemiological studies have revealed the circulation of diverse serotypes, frequent genetic recombination events, and geographically distinct patterns of spread [30,31]. In this context, continuous molecular surveillance of dominant regional serotypes is essential for understanding epidemiological dynamics and for guiding the development of effective control and vaccination strategies. Türkiye occupies a strategically important geographical position, bridging Asia and Europe, two major poultry production regions. Consequently, molecular monitoring in this region provides a unique opportunity to detect circulating variants and dynamically observe potential transboundary movements. Such surveillance efforts enable the early identification of emerging strains and support the implementation of appropriate preventive and control measures. In this context, the data generated in the present study are particularly valuable, as they contribute meaningful regional insights into the molecular epidemiology of FAdVs and enhance the broader understanding of viral circulation patterns at the intercontinental interface.

Although our work provides critical data for the commercial poultry industry, it has certain limitations. In this context, one limitation of the present study is that the phylogenetic analysis was based on a partial fragment of the hexon L1 region. Although this region is widely accepted as a reliable marker for serotype identification, analysis of additional genomic regions such as the fiber gene or full-length hexon sequences, or ideally whole-genome sequencing, would provide higher phylogenetic resolution and allow a more comprehensive assessment of the genetic diversity and evolutionary dynamics of circulating FAdV strains. Secondly, the present study is the use of pooled liver samples from multiple birds within each flock. Although pooling provides a practical approach for outbreak-level surveillance, it may mask potential intra-flock genetic diversity or the presence of co-circulating viral variants. Another limitation is the absence of histopathological examination; although the gross lesions observed at necropsy were characteristic and strongly consistent with FAdV-associated disease and thus considered sufficient for presumptive diagnosis in field conditions, definitive microscopic confirmation of inclusion body hepatitis could not be established.

The absence of licensed commercial vaccines against FAdV in Türkiye remains a major challenge for disease control. Reliance solely on biosecurity measures appears insufficient, as outbreaks occurred despite the implementation of standard management practices. The consistent detection of a single dominant serotype highlights the potential value of autogenous vaccines derived from locally circulating strains. Such vaccines have proven effective in other countries and may represent a practical strategy for reducing mortality and limiting virus transmission within commercial poultry systems.

## 5. Conclusions

The results of this study demonstrate that FAdV-8b is the predominant etiological agent associated with adenoviral disease outbreaks in broiler and layer flocks in the Southern Marmara and Aegean regions. The high genetic similarity among isolates, their clustering within a single phylogenetic lineage, and their detection across multiple provinces indicate ongoing regional circulation of a stable viral population. These findings emphasize the importance of molecular surveillance, breeder screening programs, and the development of targeted vaccination strategies to mitigate the impact of FAdV infections in commercial poultry production.

## Figures and Tables

**Table 1:** Nucleotide sequence comparison of the partial hexon gene (L1 loop region) of FAdV isolates detected in broiler and layer flocks in Türkiye with the previously reported Turkish FAdV-8b strain (MK937075.1, TR/BVKE/R/D-1) and the NCBI reference sequence (NC_014969.1). Numbers indicate nucleotide positions within the analyzed fragment. Dots (.) denote identity with the reference strain, while letters represent nucleotide substitutions.

	Position in the Gene																			
Strain	356	358	360	431	438	442	453	509	510	566	568	571	598	609	618	620	621	636	638	639
NC_014969.1	A	C	A	G	C	A	A	C	A	G	G	G	G	T	T	G	G	C	A	T
MK937075.1	.	.	C	A	G	T	G	G	G	.	.	A	.	C	A	.	C	G	G	C
FAdV_Layer_Turkiye_1	.	.	C	A	G	T	G	G	G	.	.	A	.	C	A	.	C	G	G	C
FAdV_Layer_Turkiye_2	.	.	C	A	G	T	G	G	G	.	.	A	.	C	A	.	C	G	G	C
FAdV_Broiler_Turkiye_1	.	.	C	A	G	T	G	G	G	.	.	A	.	C	A	.	C	G	G	C
FAdV_Broiler_Turkiye_2	.	.	C	A	G	T	G	G	G	.	.	A	.	C	A	.	C	A	G	C
FAdV_Broiler_Turkiye_3	.	.	C	A	G	T	G	G	G	.	.	A	.	C	A	.	C	G	G	C
FAdV_Broiler_Turkiye_4	.	.	C	A	G	T	G	G	G	A	A	A	C	C	A	T	C	A	.	C
FAdV_Broiler_Turkiye_5	.	.	C	A	G	T	G	G	G	.	.	A	.	C	A	.	C	G	G	C
FAdV_Broiler_Turkiye_6	.	.	C	A	G	T	G	G	G	.	.	A	.	C	A	.	C	G	G	C
FAdV_Broiler_Turkiye_7	.	.	C	A	G	T	G	G	G	.	.	A	.	C	A	.	C	G	G	C
FAdV_Broiler_Turkiye_9	.	.	C	A	G	T	G	G	G	.	.	A	.	C	A	.	C	G	G	C
FAdV_Broiler_Turkiye_10	C	A	C	A	G	T	G	G	G	.	.	A	.	C	A	.	C	G	G	C

**Table 2:** Deduced amino acid sequence alignment of the hexon L1 loop region of FAdV isolates from broiler and layer flocks in Türkiye in comparison with the Turkish reference strain (MK937075.1, TR/BVKE/R/D-1) and the NCBI reference sequence (NC_014969.1). Numbers represent amino acid positions within the analyzed region. Dots (.) indicate residues identical to the reference strain, while letters represent amino acid substitutions.

	Position in the Gene											
Strain	119	120	144	146	148	170	189	190	191	200	207	213
NC_014969.1	K	P	G	N	T	A	S	E	V	A	R	N
MK937075.1	.	.	E	K	S	G	.	.	M	.	.	S
FAdV_Layer_Turkiye_1	.	.	E	K	S	G	.	.	M	.	.	S
FAdV_Layer_Turkiye_2	.	.	E	K	S	G	.	.	M	.	.	S
FAdV_Broiler_Turkiye_1	.	.	E	K	S	G	.	.	M	.	.	S
FAdV_Broiler_Turkiye_2	.	.	E	K	S	G	.	.	M	.	.	S
FAdV_Broiler_Turkiye_3	.	.	E	K	S	G	.	.	M	.	.	S
FAdV_Broiler_Turkiye_4	.	.	E	K	S	G	N	K	M	P	L	N
FAdV_Broiler_Turkiye_5	.	.	E	K	S	G	.	.	M	.	.	S
FAdV_Broiler_Turkiye_6	.	.	E	K	S	G	.	.	M	.	.	S
FAdV_Broiler_Turkiye_7	.	.	E	K	S	G	.	.	M	.	.	S
FAdV_Broiler_Turkiye_9	.	.	E	K	S	G	.	.	M	.	.	S
FAdV_Broiler_Turkiye_10	T	T	E	K	S	G	.	.	M	.	.	S

## Data Availability

The original contributions presented in this study are included in the article. Further inquiries can be directed to the corresponding author.

## Abbreviations

The following abbreviations are used in this manuscript:

FAdV	Fowl adenovirus
IBH	Inclusion body hepatitis
HHS	Hepatitis-hydropericardium syndrome
AGE	Adenoviral gizzard erosion
NCBI	National Center for Biotechnology Information

## References

[1] Fitzgerald S.D., Rautenschlein S., Mahsoub H.M., Pierson F.W., Reed W.M., Jack S.W. (2020). Adenovirus infections. Diseases of Poultry.

[2] Schachner A., Matos M., Grafl B., Hess M. (2018). Fowl adenovirus-induced diseases and strategies for their control–a review on the current global situation. Avian Pathol..

[3] Fonseca A.S.K., Kipper D., Ikuta N., Lunge V.R. (2025). Molecular Characterization of Fowl Adenovirus from Brazilian Poultry Farms. Poultry.

[4] Li P., Zheng P., Zhang T., Wen G., Shao H., Luo Q. (2017). Fowl adenovirus serotype 4: Epidemiology, pathogenesis, diagnostic detection, and vaccine strategies. Poult. Sci..

[5] Schachner A., Marek A., Grafl B., Hess M. (2016). Detailed molecular analyses of the hexon loop-1 and fibers of fowl aviadenoviruses reveal new insights into the antigenic relationship and confirm that specific genotypes are involved in field outbreaks of inclusion body hepatitis. Vet. Microbiol..

[6] Sohaimi N.M., Hair-Bejo M. (2021). A recent perspective on fiber and hexon genes proteins analyses of fowl adenovirus toward virus infectivity—A review. Open Vet. J..

[7] ICTV (International Committee on Taxonomy of Viruses) Virus Taxonomy: 2023 Release. https://ictv.global/taxonomy.

[8] de Faria V.B., Silva C.C., de Paula Damaso P., Savoldi I.R., Sommerfeld S., Fonseca B.B. (2025). Epidemiological insights into fowl adenovirus, astrovirus, and avian reovirus in Brazilian poultry flocks: A cross-sectional study. Poult. Sci..

[9] Song Y., Liu L., Sun W., Gao W., Song X., Wang Y., Wei Q., Huang Z., Li X. (2024). Identification, pathogenicity and molecular characterization of a novel fowl adenovirus 8b strain. Poult. Sci..

[10] Sahindokuyucu I., Yilmaz Cagirgan O., Kilic H., Cagirgan A., Yazici Z. (2025). Research on molecular epidemiology of aviadenovirus in Turkish commercial poultry flocks. Br. Poult. Sci..

[11] Mase M., Mitake H., Inoue T., Imada T. (2009). Identification of group I–III avian adenovirus by PCR coupled with direct sequencing of the hexon gene. J. Vet. Med. Sci..

[12] Fu Y., Lou M., Sun J., Liu S., Wang T., Wang F., Wang Y., Yu K., Li Y., Liu A. (2026). Characterization of a Natural Recombinant Fowl Adenovirus of Serotypes 8a and 8b. Poult. Sci..

[13] Huang Q., Ma X., Huang X., Huang Y., Yang S., Zhang L., Cui N., Xu C. (2019). Pathogenicity and complete genome sequence of a fowl adenovirus serotype 8b isolate from China. Poult. Sci..

[14] Liu A., Zhang Y., Wang J., Cui H., Qi X., Liu C., Zhang Y., Li K., Gao L., Wang X. (2022). Complete genome analysis and animal model development of fowl adenovirus 8b. Viruses.

[15] Kardoudi A., Benani A., Allaoui A., Kichou F., Biskri L., Ouchhour I., Fellahi S. (2025). Fowl adenovirus serotype 1: From gizzard erosion to comprehensive insights into genome organization, epidemiology, pathogenesis, diagnosis, and prevention. Vet. Sci..

[16] Ouchhour I., Fellahi S., Khantour A.E., Darkaoui S., Mouahid M., Touzani C.D., Abghour S., Kichou F. (2025). Fowl aviadenoviruses in Moroccan poultry: Pathological characteristics and phylogenetic analysis of circulating fowl aviadenovirus strains from 2012 to 2024. Avian Pathol..

[17] Şahindokuyucu İ., Çöven F., Kılıç H., Yılmaz Ö., Kars M., Yazıcıoğlu Ö., Ertunç E., Yazıcı Z. (2020). First report of fowl aviadenovirus serotypes FAdV-8b and FAdV-11 associated with inclusion body hepatitis in commercial broiler and broiler-breeder flocks in Turkey. Arch. Virol..

[18] Cizmecigil U.Y., Umar S., Yilmaz A., Bayraktar E., Turan N., Tali B., Aydin O., Tali H.E., Yaramanoglu M., Yilmaz S.G. (2020). Characterisation of fowl adenovirus (FAdV-8b) strain concerning the geographic analysis and pathological lesions associated with inclusion body hepatitis in broiler flocks in Turkey. J. Vet. Res..

[19] Zhang Y., Liu A., Wang Y., Cui H., Gao Y., Qi X., Liu C., Zhang Y., Li K., Gao L. (2021). A single amino acid at residue 188 of the hexon protein is responsible for the pathogenicity of the emerging novel virus fowl adenovirus 4. J. Virol..

[20] Sadekuzzaman M., Miah M.S., Parvin R., Haque M.E., Islam T.R., Sigma S.H., Hossain M.G., Hayat S., Hossain M.T., Islam M.A. (2024). Pathological investigation, molecular characterization and first-time isolation of the predominant serotypes of fowl adenovirus (FAdV-D and E) from commercial poultry in Bangladesh. Front. Microbiol..

[21] Oliver-Ferrando S., Dolz R., Calderón C., Valle R., Rivas R., Pérez M., Biarnés M., Blanco A., Bertran K., Ramis A. (2017). Epidemiological and pathological investigation of fowl aviadenovirus serotypes 8b and 11 isolated from chickens with inclusion body hepatitis in Spain (2011–2013). Avian Pathol..

[22] Okuda Y., Ono M., Shibata I., Sato S. (2004). Pathogenicity of serotype 8 fowl adenovirus isolated from gizzard erosions of slaughtered broiler chickens. J. Vet. Med. Sci..

[23] McFerran J., Smyth J. (2000). Avian adenoviruses. Rev. Sci. Tech. (Int. Off. Epizoot.).

[24] Grgic H., Philippe C., Ojkic D., Nagy É. (2006). Study of vertical transmission of fowl adenoviruses. Can. J. Vet. Res..

[25] Chitradevi S. (2025). Fowl Adenovirus Infection in Chicken: A Comprehensive Review. Microbiol. Res. J. Int..

[26] Sohaimi N.M., Clifford U.C. (2021). Fowl adenovirus in chickens: Diseases, epidemiology, impact, and control strategies to the Malaysian poultry industry–A review. J. World’s Poult. Res..

[27] Islam M.M., Nadia M.M.A., Islam M.R., Islam M.S., Sunny S.A., Islam M., Sultana S., Alam M.J. (2026). Fowl Adenovirus Infections: A Comprehensive Review of Prevalence, Pathogenesis, Diagnosis, Control, and Economic Impact. Poult. Sci..

[28] Franzo G., Prentza Z., Paparounis T., Tsiouris V., Centonze G., Legnardi M., Catelli E., Tucciarone C.M., Koutoulis K., Cecchinato M. (2020). Molecular epidemiology of fowl adenoviruses in Greece. Poult. Sci..

[29] Niczyporuk J.S. (2018). Deep analysis of Loop L1 HVRs1-4 region of the hexon gene of adenovirus field strains isolated in Poland. PLoS ONE.

[30] Pan Q., Liu L., Gao Y., Liu C., Qi X., Zhang Y., Wang Y., Li K., Gao L., Wang X. (2017). Characterization of a hypervirulent fowl adenovirus 4 with the novel genotype newly prevalent in China and establishment of reproduction infection model of hydropericardium syndrome in chickens. Poult. Sci..

[31] Kiss I., Homonnay Z., Mató T., Bányai K., Palya V. (2021). Research Note: An overview on distribution of fowl adenoviruses. Poult. Sci..

